# The Impact of Post-bariatric Abdominoplasty on Secondary Weight Regain After Roux-en-Y Gastric Bypass

**DOI:** 10.3389/fendo.2020.00459

**Published:** 2020-07-30

**Authors:** Jorunn Sandvik, Torstein Hole, Christian Klöckner, Bård Kulseng, Arne Wibe

**Affiliations:** ^1^Clinic of Medicine and Rehabilitation, Møre and Romsdal Hospital Trust, Alesund, Norway; ^2^Department of Surgery, Center for Obesity, St. Olav Hospital, Trondheim University Hospital, Trondheim, Norway; ^3^Obesity Research Group, Department of Clinical and Molecular Medicine, Norwegian University of Science and Technology, Trondheim, Norway; ^4^Faculty of Medicine and Health Sciences, Norwegian University of Science and Technology, Trondheim, Norway; ^5^Department of Psychology, NTNU - Norwegian University of Science and Technology, Trondheim, Norway; ^6^Department of Clinical and Molecular Medicine, Norwegian University of Science and Technology, Trondheim, Norway; ^7^Department of Surgery, St. Olav Hospital, Trondheim University Hospital, Trondheim, Norway

**Keywords:** post-bariatric abdominoplasty, bariatric surgery, gastric bypass (RYGB), post-bariatric weight regain, post-bariatric body contouring

## Abstract

Roux-en-Y gastric bypass (RYGB), implies a considerable weight loss during the first two years after surgery. Excess skin due to rapid weight loss might affect self-esteem, decrease quality of life and be a hindrance to physical activity. Removing excess skin might reduce secondary weight regain. Among plastic surgeons, a BMI <30 kg/m^2^ is usually required to have abdominoplasty (AP). Many RYGB patients never reach this threshold even if they have a considerable weight loss and experience practical as well as emotional problems due to excess skin. The aim of this study was to investigate the role of abominoplasty on weight development until five years, among patients who did and did not achieve a nadir BMI <30 kg/m^2^ during the first two years after RYGB. Data on 645 patients from a single center RYGB-quality register from 2004 to 2013 with baseline and follow-up data were analyzed. Post-bariatric AP was publicly funded if medically needed. Weight regain (WR) from nadir weight to five years was analyzed as percentage WR of maximal weight loss. Nadir BMI was available in 633 (98.1%) patients, and BMI after five years in 553 (85.7%) patients. The 233 patients with nadir BMI <30 kg/m^2^ who underwent AP regained 17.8 (±16.1) % of their maximal weight loss at five years compared to 24.2 (±19.7) % in 185 patients not having AP (*p* < 0.001). The 27 patients with nadir BMI > 30 kg/m^2^ within two years after RYGB who underwent AP regained 12.9 (±19.3) % compared to 31.4 (±24.7) % in 188 patients without AP (*p* < 0.001). This procedure was more common among women than men, as 224 (46.4%) women, and 36 (22.2%) men underwent AP. Abdominoplasty was associated with reduced secondary weight regain after RYGB in this study. Whether this is caused by increased bodily satisfaction and better physical function, or a biological response to reduction of adipose tissue remains unclear. If removing abdominal subcutaneous adipose tissue prevent secondary weight regain and increase the robustness of bariatric surgery, this should be offered as part of the standard treatment after bariatric surgery.

## Introduction

Surgical procedures for treatment of severe obesity and obesity-related comorbidities have been integrated as part of public as well as private health care during the last two decades. The term *bariatric surgery*, with focus on weight reduction, has to a certain extent been replaced by the term *metabolic surgery*, with focus on the improvement of type 2 diabetes mellitus (T2DM), cardiovascular disease, and other diseases where inflammation plays a role. Worldwide, more than half a million people have bariatric surgery every year, and most of them experience considerable weight loss during the first years after surgery (1, 2). Usually the nadir weight is achieved between 1 and 2 years after surgery; however, most patients experience a varying degree of weight regain (WR) within the following years (3). Secondary WR after bariatric surgery might be regarded as a failure of the surgical method or as a natural progression of the chronic disease of obesity.

Many factors may contribute to the secondary WR, and the patients' motivation for lifelong changes in diet and habits of physical activity has been regarded as the main factor for weight loss maintenance after bariatric surgery. However, the knowledge on the biological mechanism beyond voluntary control of secondary WR after bariatric surgery is increasing (4).

One of the factors that seem to be associated with the degree and durability of weight loss is post-bariatric body contouring procedures (5–7). For some patients, considerable weight loss after bariatric surgery implies burdensome excess skin mainly on abdomen, but also on thighs, arms, breast, back, and face. The excess skin might result in intertrigo, skin infections, and mobility problems, as well as a negative body image, depression, and social dysfunction (8, 9).

When body contouring procedures are performed, excess skin and the adjacent subcutaneous adipose tissue are resected. Improvements in quality of life and bodily function after body contouring surgery have been related to psychological factors and improved self-esteem (10). However, metabolic consequences of removing excess subcutaneous adipose tissue by body contouring procedures have to a lesser extent been explored.

During the last years, an increasing number of the biological functions of adipose tissue have been revealed. Contrary to former beliefs, the adipose tissue is a dynamic and metabolic active organ, secreting various hormones and cytokines involved in appetite regulation, energy metabolism, and inflammation (11, 12). It is therefore relevant to question if removing excess skin and subcutaneous tissue has a beneficial biological effect on reducing secondary WR after bariatric surgery.

Studies on the metabolic effect of removing subcutaneous tissue by liposuction or lipectomy in people with obesity who did not undergo bariatric surgery have shown a reduction in inflammation, improvement in lipid profile, normalization of glucose and insulin levels, and improvement in cardiac function (13–15). In contrast, a meta-analysis on abdominal lipectomy in non-bariatric women from 2015 did not reveal significant effects on metabolic syndrome or insulin sensitivity, and only a short-term effect on body fat and weight (16, 17).

Bariatric surgery is performed in most countries in the world and in health care systems with various financial models. The access to bariatric surgery is limited by capacity as well as financial coverage for the patients. Post-bariatric body contouring surgery is even less accessible, and it may not be covered by public health service or private health insurance (18).

Among 37,906 patients who underwent bariatric surgery in New York from 2004 to 2010, <6% underwent an abdominoplasty during the following years (19). This low utilization of post-bariatric abdominoplasty was mainly explained by financial reasons.

Abdominoplasty is the most common post-bariatric body contouring procedure, but excess skin on thighs, arms, and upper truncus may also be a problem in need of surgical treatment (20). Most plastic surgeons have an upper body mass index (BMI) threshold of 28 or 30 kg/m^2^ to perform abdominoplasty, because of the assumed increased risk of complications with higher BMI (21). Because only half of the patients undergoing bariatric surgery reach this BMI threshold, patients in the upper BMI levels before bariatric surgery may struggle with excess skin after a massive weight loss without any treatment alternatives. For them, the physical, mental, and social impairment of excess skin after bariatric surgery may imply an increase in secondary WR. Hence, the role of excess subcutaneous fat in secondary WR needs to be investigated.

Secondary WR after initial successful post-bariatric weight loss has been calculated in different ways; measuring WR as percentage of maximal weight loss is best associated with changes in clinical outcomes after bariatric surgery (22).

The aim of this study was to explore the role of abdominoplasty on weight development from the time of maximum weight loss until 5 years after roux-en-Y gastric bypass (RYGB), in a population who had access to abdominoplasty 2 years after surgery if they reached a nadir BMI of <30 kg/m^2^, and there was a medical indication for the procedure.

## Materials and Methods

This study is a retrospective analysis of prospectively collected data on 645 adult patients who underwent laparoscopic RYGB from 2004 to 2013 as a primary treatment for severe obesity at Ålesund Hospital, a public hospital in Western Norway. Roux-en-Y gastric bypass was a treatment option for patients aged 18–60 years, with BMI >40 kg/m^2^ or >35 kg/m^2^ with obesity-related comorbidity. Details on the surgical method have been published in a previous article (23).

All patients who underwent RYGB at the hospital from 2004 were registered in a local quality registry, and data on weight changes, selected laboratory tests, body contouring procedures, and other relevant events related to the RYGB procedure were registered at baseline and at follow-up visits 2, 6, 12, 18, 24, 36, 48, and 60 months after surgery. Nadir BMI was the lowest BMI measured at the planned visits at 12, 18, or 24 months. Patients followed up for 5 years or more by the end of 2018 were included in the study.

Patients who wanted abdominoplasty or other body contouring procedures were referred to the plastic surgeon for assessment 18 months after RYGB if they had a BMI of <30 kg/m^2^. In general, the patients had to wait at least 2 years after RYGB before they could have abdominoplasty, and the weight had to be stable during 6 months before surgery.

The abdominoplasty was not performed at the same hospital as the RYGB, but at private or public hospitals in the same region. The abdominoplasty was publicly funded regardless of where the operation was performed, if there was a medical indication for surgical treatment. Reports from the plastic surgeon were in most cases sent to the study hospital, and the date of the procedures was added to the quality registry. If reported, the amount of removed tissue and surgical technique were also registered.

Weight regain from nadir weight to 5 years after RYGB was analyzed as percentage WR of maximal weight loss, and WR in kilograms.

Continuous variables are given as means ± standard deviation (SD) if normally distributed, and as median and interquartile range (IQR) if non-normally distributed. Categorical variables are reported in numbers and percentages. Independent *t*-tests were performed for normally distributed continuous variables, and non-parametric test for non-normally distributed variables. χ^2^-tests were performed for categorical variables. Differences were considered significant at *P* < 0.05.

## Results

From a total of 645 patients, 483 (75%) women, and 162 (25%) men, information on nadir BMI was available in 633 patients (98.1%), and BMI 5 years after RYGB was available in 553 patients (85.7%). Four patients had died within 5 years after RYGB, and 15 (patients 2.3%) had moved out of the region before 5 years. Details on comorbidities at baseline and T2DM after 5 years are given in Table 1.

**Table 1:** Comorbidities at baseline and T2DM status 5 years after RYGB in patients who achieved nadir BMI greater than or less than 30 kg/m^2^, with and without abdominoplasty.

	**Nadir BMI <30 kg/m**^****2****^ ***n*** **=** **418**		***P***	**Nadir BMI>30 kg/m**^****2****^ ***n*** **=** **210**		***P***
	**With AP *n* = 233**	**Without AP *n* = 185**		**With AP *n* = 27**	**Without AP *n* = 183**	
T2DM at baseline	67 (16%)			38 (18.1%)		
	28 (12.0%)	39 (21.2%)	*P* = 0.01	3 (11.55)	34 (18.5%)	*P* = 0.379
T2DM at 5 years	13 (3.1%)			10 (4.8%)		
	5 (2.1%)	8(4.3%)	*P* = 0.203	0	10 (5.5%)	n.a.
Hypertension, baseline	105 (25.1%)			62(29.5%)		
	45 (19.3%)	60 (32.6%)	*P* = 0.002	3 (11.5%)	59 (32.2%)	*P* = 0.031
Sleep apnea baseline	95 (22.7%)			59 (28.4%)		
	45(19.4%)	50(27.2%)	*P* = 0.062	9 (33.3%)	51(27.9%)	*P* = 0.557
Hyperlipidemia baseline	52 (12.4%)			29 (13.8%)		
	18 (7.7%)	34 (18.4%)	*P* = 0.001	2 (7.4%)	27 (14.6%)	*P* = 0.325

AP, abdominoplasty; T2DM, type 2 diabetes mellitus.

Within 2 years after RYGB, a nadir BMI of 30 kg/m^2^ or less was achieved by 418 patients (66.5%), as 331 (68.5%) of the women and 87 (53.7%) of the men reached this threshold (*P* < 0.001). There was no difference in age among patients with nadir BMI greater than or less than 30 kg/m^2^, with mean age of 40 (±9.7) years.

**Figure 1:**
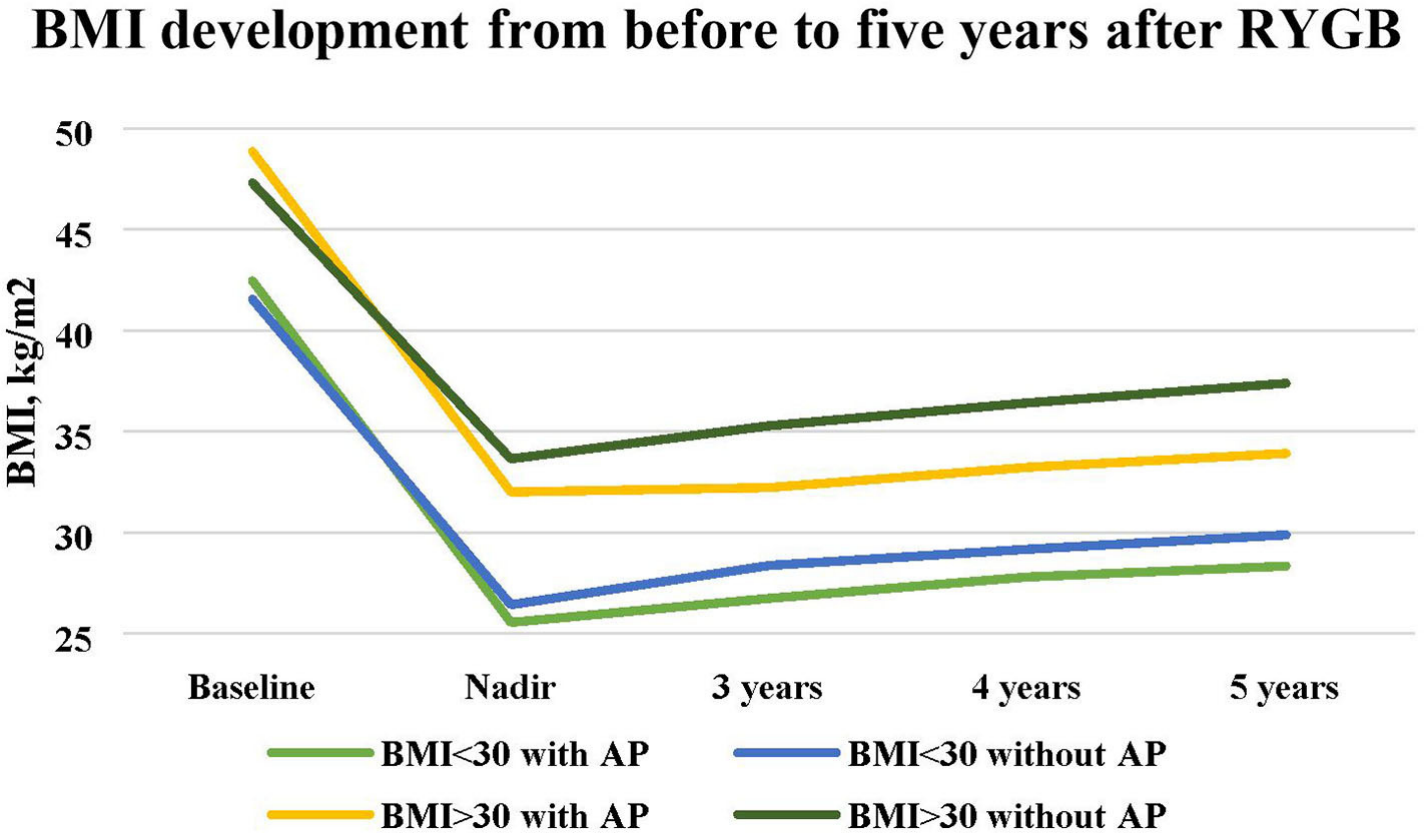
Changes in mean BMI from baseline to 5 years after RYGB in patients with and without abdominoplasty and Nadir BMI above or below 30 kg/m^2^.

When comparing the patients with nadir BMI greater than or less than 30 kg/m^2^, the patients who achieved BMI 30 kg/m^2^ or less had a mean preoperative BMI of 42.0 (±3.6) kg/m^2^, compared to 47.4 (±5.4) kg/m^2^ in the patients who did not achieve a nadir BMI of less than this limit. Mean nadir BMI in the lower BMI group was 26.0 (±2.2) kg/m^2^, compared to 33.4 (±2.8) kg/m^2^, and mean BMI at 5 years was 29.0 (±3.5) kg/m^2^ compared to 36.9 (±4.4) kg/m^2^. The differences were significant at all points (*P* < 0.001) (Figure 1).

The mean percentage of total weight loss (%TWL) at nadir and after 5 years was 38.2% (±6.3%) and 30.5% (±9.0%) for the patients with nadir BMI of <30 kg/m^2^, compared to 29.4% (±7.1%) and 21.3% (±9.6%) for the patients with nadir BMI of >30 kg/m^2^ (*P* < 0.001).

Secondary WR measured as percentage of maximal weight loss from nadir to 5 years was 28.9% (±24.8%) in patients with nadir BMI of >30 kg/m^2^ and 20.4% (± 18.0%) in all of the patients with nadir BMI below 30 kg/m^2^ (*P* < 0.001).

Among the 418 patients who achieved BMI ≤ 30 kg/m^2^, there were 233 patients (55.7%) who underwent abdominoplasty. The procedure was more common among women than men, as 202 (61.0%) of the women and 31 (35.6%) of the men with BMI ≤ 30 kg/m^2^ underwent abdominoplasty (*P* < 0.001). The patients who had abdominoplasty were 39 (±8.8) years compared to 40.6 (±10.7) years in the patients who did not (ns). The median (IQR) time from RYGB to abdominoplasty was 31.0 (25.5–37) months.

In patients with nadir BMI of 30 kg/m^2^ or less, preoperative BMI, nadir BMI, and BMI at 5 years were 42.4 (±3.7) kg/m^2^, 25.6 (±2.0) kg/m^2^, and 28.5 (±3.2) kg/m in the abdominoplasty group, and 41.6 (±3.5) kg/m^2^, 26.4 (±2.3) kg/m^2^, and 30.1 (±3.7) kg/m^2^ among those who did not have abdominoplasty (*P* < 0.001 at all points) (Table 2).

**Table 2:** Patients' characteristics and weight changes.

	**Nadir BMI** ** <30 kg/m**^****2****^ ***n*** **=** **418**		***P***	**Nadir BMI** **>30 kg/m**^****2****^ ***n*** **=** **210**		***P***
	**With AP *n* = 233**	**Without AP *n* = 185**		**With AP *n* = 27**	**Without AP *n* = 183**	
Age (±SD), years	39.2 (± 8.8)	40.6 (±10.7)	*P* = 0.125	36.6 (±8.2)	40.5 (±9.9)	*P* = 0.61
Female/male ratio	202/31	129/56	*P* < 0.001	22/5	118/65	*P* = 0.100
Baseline BMI (±SD) kg/m^2^, *N*	42.4 (±3.7) 233	41.6 (±3.5) 185	*P* = 0.01	48.9 (±6.0) 27	47.3 (±5.3) 183	*P* = 0.172
Nadir BMI (±SD) kg/m^2^, *n*	25.6 (±2.0) 233	26.4 (±2.3) 185	*P* < 0.001	32.0 (±2.0) 27	33.6 (±2.9) 183	*P* < 0.001
BMI 3 years after RYGB (±SD) kg/m^2^, *n*	26.7 (±2.5) 220	28.4 (±3.2) 164	*P* < 0.001	32.2 (±2.7) 22	35.3 (±3.6) 141	*P* < 0.001
BMI 4 years after RYGB (±SD) kg/m^2^, *n*	27.8 (±2.9) 212	29.2 (±3.4) 145	*P* < 0.001	33.2 (±2.7) 22	36.4 (±3.9) 145	*P* < 0.001
BMI 5 years after RYGB (±SD) kg/m^2^, *n*	28.5 (±3.2) 212	30.1 (±3.7) 156	*P* < 0.001	33.9 (±3.2) 24	37.4 (±4.3) 152	*P* < 0.001
% TWL at nadir %, *n*	39.8 (±5.7) 233	36.3 (±6.4) 184	*P* < 0.001	33.8 (±7.2) 26	28.8 (±6.9) 183	*P* < 0.001
% TWL after 3 years %, *n*	36.5 (±7.3) 220	31.3 (±7.6) 164	*P* < 0.001	33.7 (±8.7) 22	24.3 (±8.1) 141	*P* < 0.001
% TWL after 4 years %, *n*	34.1 (±7.9) 212	29.1 (±8.0) 145	*P* < 0.001	31.0 (±8.0) 22	22.2 (±8.3) 145	*P* < 0.001
% TWL after 5 years %, *n*	32.7 (±8.1) 212	27.6 (±9.1) 156	*P* < 0.001	30.0 (±8.9) 24	20.0 (±9.0) 152	*P* < 0.001
% EWL at nadir %, *n*	97.6 (±11.9) 233	92.0 (±14.7) 185	*P* < 0.001	69.7 (±8.3) 26	61.1 (±10.9) 183	*P* < 0.001
% EWL after 3 years %, *n*	89.2 (±15.3) 220	79.4 (±18.9) 164	*P* < 0.001	68.1 (±11.9) 22	51.6 (±14.1) 141	*P* < 0.001
% EWL after 4 years %, *n*	83.4 (±17.4) 212	74.3 (±19.9) 145	*P* < 0.001	63.5 (±12.0) 22	46.9 (±15.1) 145	*P* < 0.001
% EWL after 5 years %, n^**^	80.2 (±18.9) 212	69.5 (±21.6) 156	*P* < 0.001	60.9 (±14.2) 24	42.6 (±17.5) 152	*P* < 0.001
WR from nadir to 5 years after RYGB (SD) kg, n^**^	8.4 (±7.3) 212	10.7 (±8.6) 156	*P* < 0.05	6.1 (±8.5) 24	11.9 (±9.1) 152	*P* < 0.001
% WR from nadir to 5 years after RYGB of maximal weight loss, %, *n*	17.8 (±16.1) 212	24.2 (±19.7) 156	*P* = 0.001	12.9 (±19.2) 24	31.4 (±24.7) 152	*P* < 0.001

AP, abdominoplasty;

** *WR, weight regain*.

**Figure 2:**
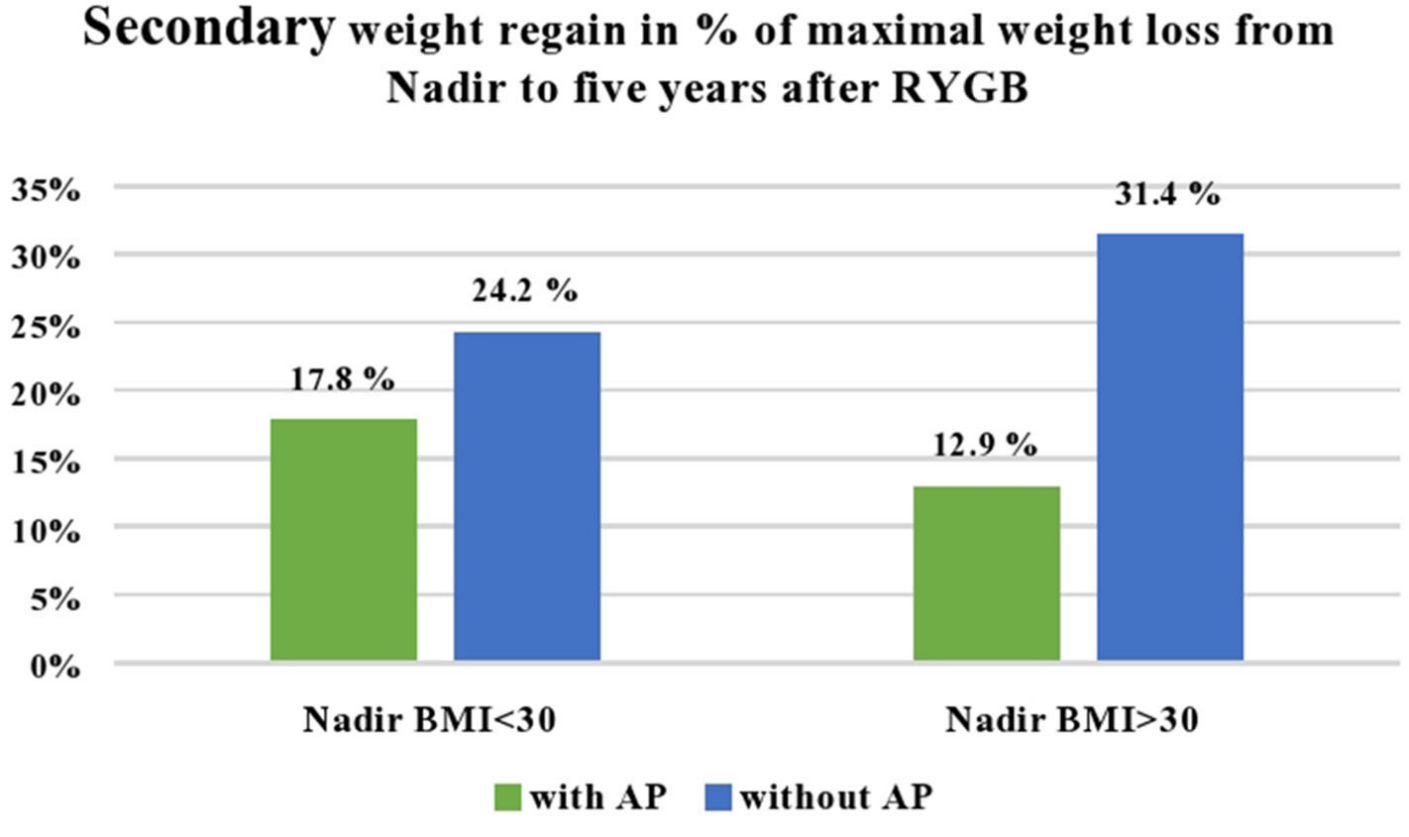
Secondary weight regain from nadir to 5 years after Roux-en-Y gastric bypass in percent of maximal weight loss from surgery to nadir weight. The number of patients who underwent abdominoplasty was 233 out of 418 patients with nadir BMI <30 kg/m^2^, and 27 out of 215 patients with BMI > 30 kg/m^2^.

From nadir to 5 years, the patients who achieved a nadir BMI of 30 kg/m^2^ or less and underwent abdominoplasty in this period regained 17.8% (±16.1%) of their maximal weight loss compared to 24.2% (±19.7%) in patients who did not have abdominoplasty (*P* < 0.001) (Figure 2). When WR was measured in kilograms, the patients who had abdominoplasty had an increase of 8.4 (±7.3) kg, compared to 10.7 (±8.6) kg in the patients who did not have abdominoplasty (*P* < 0.05).

**Figure 3:**
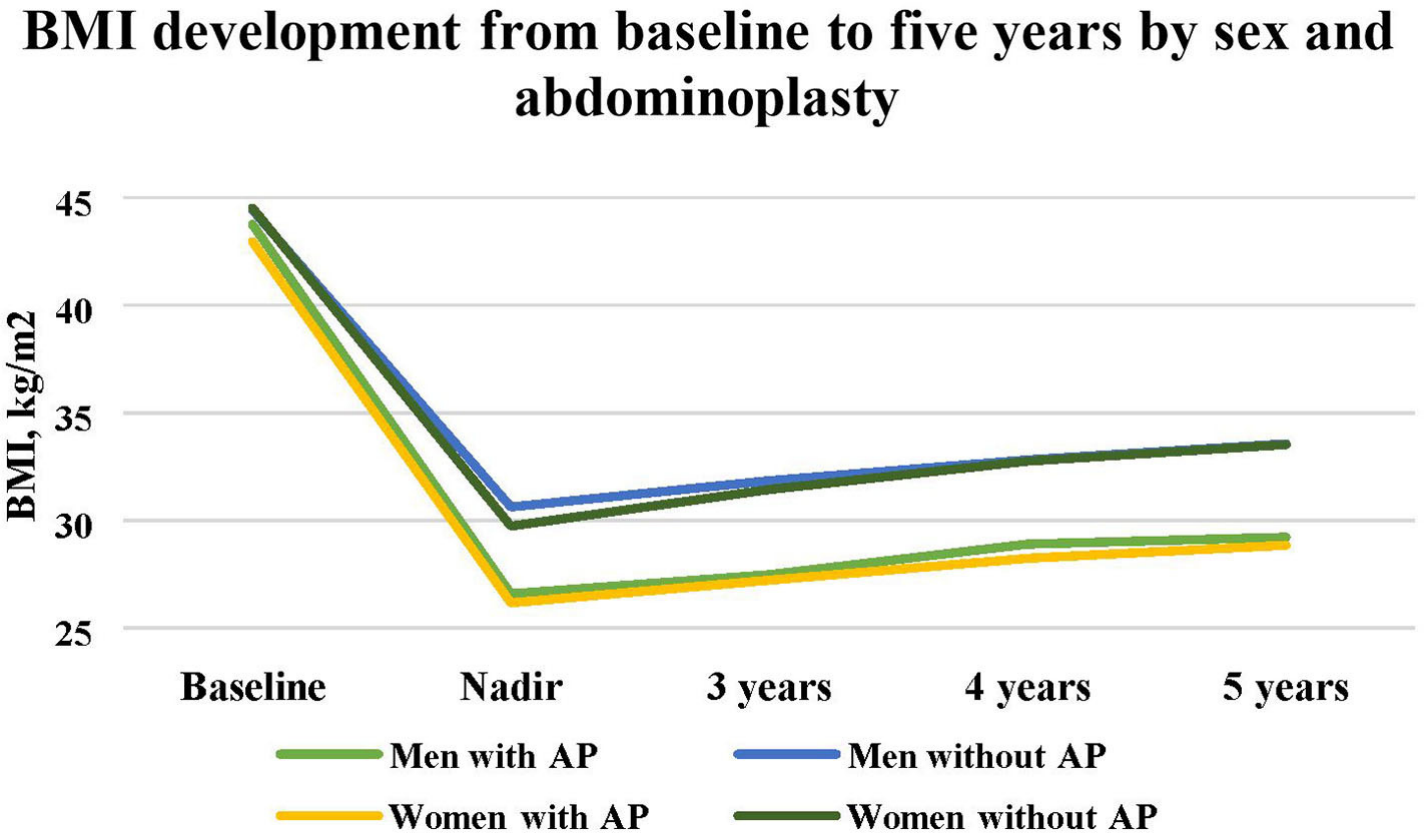
Changes in mean BMI from baseline until 5 years after RYGB in men and women with and without abdominoplasty.

Abdominoplasty was mainly performed in the patients who achieved BMI of <30 kg/m^2^ during the first 2 years after RYGB. However, from the 215 patients with a nadir BMI of >30 kg/m^2^ during the first 2 years after RYGB, there were 27 (12.5%) patients, 22 women and 5 men, who had abdominoplasty at a median 35.5 (30.5–54.7) months after RYGB (Table 2). The secondary WR in this group was 6.1 (± 8.5) kg compared to 11.9 (±9.1) kg (*P* < 0.001), or measured as WR in percentage of maximal weight loss 12.9% (±19.3%) compared to 31.4% (±24.7%) in the patients with nadir BMI of >30 kg/m^2^ who did not undergo abdominoplasty (*P* < 0.001) (Figure 2). Men and women who underwent abdominoplasty had similar weight development (Figure 3).

When it comes to other body contouring procedures, 2 men and 48 women had breast corrections (reduction, lift, or augmentation). Also, 2 men and 43 women had excess skin on thighs removed, and 1 man and 29 women had plastic surgery on upper arms in the follow-up period. These procedures were performed at a later stage after RYGB and are not analyzed in relation to secondary WR in this study. Of all patients, 198 (30.7%) had one body contouring procedure, 52 (8.1%) had two procedures, and 25 (3.9%) had more than two procedures.

The reports from the plastic surgeons contained information on the amount of tissue removed at the abdominoplasty in 145 (55.8%) of 260 cases, the mean weight being 1.76 (±0.97) kg. As this information was not available for all patients who had abdominoplasty, individual values on excised tissue have not been implemented in the analyses of secondary WR in this study. However, a correlation between weight of excised tissue and secondary WR was not found in the subgroup with available data (*P* = 0.737).

## Discussion

In the present study, less secondary WR was found in the patients who underwent abdominoplasty after RYGB, and the difference was highest in the group with nadir BMI of >30 kg/m^2^ within the first 2 years after RYGB. The number of patients in this group was low, and there was no information on BMI at time of abdominoplasty.

More women than men had abdominoplasty, as there were more women who achieved a BMI of <30 kg/m^2^ within the first 2 years after RYGB. Moreover, the interest for body contouring surgery after bariatric surgery were higher among women than men, as has been found in other studies (24–26).

An assumed higher risk of post-operative complications has been the reason for limiting abdominoplasty after major weight loss to patients with BMI of <30 kg/m^2^ (27). However, there are studies that did not find any difference in complications among patients with BMI greater than or less than 30 kg/m^2^ undergoing post-bariatric body contorting surgery (28). In the present study, the abdominoplasties were mainly performed as day surgery in private hospitals without facilities for postoperative observation in a surgical ward. Furthermore, patients with higher surgical risk due to comorbidities or higher BMI had less access to treatment due to limited capacity in the public hospitals in the region.

A study by de Vries et al. including 126 patients who underwent post-bariatric body contouring surgery found that there might be an association between body contouring surgery and weight loss maintenance after 3 and 4 years, but body contouring surgery was not associated with maintenance of achieved improvement of comorbidities (29). Two other cohort studies have reported less WR if body contouring procedures were performed after RYGB; the weight developments described in these studies were on the same level as in the present study (6, 7).

When it comes to the metabolic and biochemical modifications occurring after abdominoplasty in patients with BMI between 30 and 35 kg/m^2^ who had not undergone bariatric surgery, a study by Cuomo et al. revealed an increase in adiponectin levels and a significant decrease in BMI at 1 year after abdominoplasty, leading the authors to suggest abdominoplasty or liposuction as a method for improvement of insulin sensitivity in people with moderate obesity (13). Several studies have indicated a significant reduction in cardiovascular risk score after large volume liposuction or abdominoplasty, at a level comparable to bariatric surgery (14). These findings might implicate that patients who have undergone bariatric surgery have an additional beneficial metabolic effect of having abdominoplasty.

To our knowledge the role of body contouring procedures has not been emphasized when the favorable long-term effect of bariatric surgery on metabolic health and reduction in morbidity and mortality are presented. It is timely to ask if some of the effects attributed to bariatric surgery in fact are caused by removing adipose tissue in the period after the bariatric procedure.

More knowledge on the metabolic effects of plastic surgery in patients with obesity may lead to new treatment algorithms including abdominoplasty or liposuction as part of a multimodal program including non-surgical weight loss treatment, drugs, and plastic surgery, as well as bariatric surgery. As acceptance of obesity as a chronic relapsing disease worth treatment increases among patients as well as in the medical profession, more tools are needed to provide patients with the best treatment options in the various stages of the disease.

## Strengths and Limitations

Compared to other studies that have found a beneficial effect of abdominoplasty on secondary WR after bariatric surgery, the present study had a higher number of patients and a higher percentage of patients undergoing abdominoplasty as the treatment was covered by public means. The data were collected prospectively, and the present cohort had a high rate of follow-up and an observation time of at least 5 years after RYGB.

A limitation in the present study is that data were collected in a clinical setting, and details related to technical aspects of the abdominoplasty were not registered. The reports from the plastic surgeons were not standardized, and information on the amount of tissue removed during abdominoplasty was available for only half of the patients. Because of limitations in the data available, changes in metabolic health or inflammation could not be documented by laboratory tests or clinical measurements before and after abdominoplasty.

## Conclusion

Abdominoplasty was associated with reduced secondary WR after RYGB in this study, and particularly among the patients who did not achieve a nadir BMI of <30 kg/m^2^ during the first 2 years after surgery. Whether this is caused by increased bodily satisfaction and better physical function or a biological response to reduction of adipose tissue remains unclear. If removing subcutaneous adipose tissue from abdomen prevents secondary WR and increases the robustness of bariatric surgery, this should be offered as part of the standard treatment after bariatric surgery.

The clinical relevance of the difference in secondary WR was not explored in this study, and the biological role of abdominoplasty on weight development after bariatric surgery remains an unanswered question.

## Data Availability Statement

The datasets generated for this study will not be made publicly available due to national legislation on local quality registries at public hospitals.

## Ethics Statement

The studies involving human participants were reviewed and approved by the Regional Ethics Committee, REK sør-øst, Norway. Written informed consent for participation was not required for this study in accordance with the national legislation and the institutional requirements.

## Author Contributions

JS collected the data. Analyses were performed by JS and CK. The conceptual framework was developed in discussion among all authors. JS wrote the first manuscript. All authors contributed to the final version of the manuscript.

## Conflict of Interest

The authors declare that the research was conducted in the absence of any commercial or financial relationships that could be construed as a potential conflict of interest.

## References

[1] Angrisani L Santonicola A Iovino P Vitiello A Higa K Himpens J. IFSO worldwide survey 2016: primary, endoluminal, and revisional procedures. Obes Surg. (2018) 28:3783–94. 10.1007/s11695-018-3450-2 30121858

[2] Welbourn R Hollyman M Kinsman R Dixon J Liem R Ottosson J. Bariatric surgery worldwide: baseline demographic description and one-year outcomes from the fourth IFSO global registry report 2018. Obes Surg. (2018). 10.1007/s11695-018-3593-1 30421326

[3] Keith CJ Jr Gullick AA Feng K Richman J Stahl R Grams J. Predictive factors of weight regain following laparoscopic Roux-en-Y gastric bypass. Surg Endosc. (2018) 32:2232–8. 10.1007/s00464-017-5913-2 29067574

[4] Karmali S Brar B Shi X Sharma AM de Gara C Birch DW. Weight recidivism post-bariatric surgery: a systematic review. Obes Surg. (2013) 23:1922–33. 10.1007/s11695-013-1070-4 23996349

[5] Froylich D Corcelles R Daigle CR Aminian A Isakov R Schauer PR. Weight loss is higher among patients who undergo body contouring procedures after bariatric surgery. Surg Obes Relat Dis. (2016) 12:1731–6. 10.1016/j.soard.2015.09.009 26723561

[6] Balagué N Combescure C Huber O Pittet-Cuénod B Modarressi A. Plastic surgery improves long-term weight control after bariatric surgery. Plast Reconstr Surg. (2013) 132:826–33. 10.1097/PRS.0b013e31829fe531 24076675

[7] Smith OJ Hachach-Haram N Greenfield M Bystrzonowski N Pucci A Batterham RL. Body contouring surgery and the maintenance of weight-loss following Roux-En-Y gastric bypass: a retrospective study. Aesthet Surg J. (2018) 38:176–82. 10.1093/asj/sjx170 29040424

[8] Biorserud C Olbers T Fagevik Olsen M. Patients' experience of surplus skin after laparoscopic gastric bypass. Obes Surg. (2011) 21:273–7. 10.1007/s11695-009-9849-z 19455374

[9] Elander A Biorserud C Staalesen T Ockell J Fagevik Olsen M. Aspects of excess skin in obesity, after weight loss, after body contouring surgery and in a reference population. Surg Obes Relat Dis. (2019) 15:305–11. 10.1016/j.soard.2018.10.032 30638792

[10] Song P Patel NB Gunther S Li CS Liu Y Lee CY. Body image & quality of life: changes with gastric bypass and body contouring. Ann Plast Surg. (2016) 76(Suppl 3):S216–21. 10.1097/SAP.0000000000000788 27070678 PMC4833608

[11] Fruhbeck G Busetto L Dicker D Yumuk V Goossens GH Hebebrand J. The ABCD of obesity: an EASO position statement on a diagnostic term with clinical and scientific implications. Obes Facts. (2019) 12:131–6. 10.1159/000497124 30844811 PMC6547280

[12] Haczeyni F Bell-Anderson KS Farrell GC. Causes and mechanisms of adipocyte enlargement and adipose expansion. Obes Rev. (2018) 19:406–20. 10.1111/obr.12646 29243339

[13] Cuomo R Russo F Sisti A Nisi G Grimaldi L Brandi C. Abdominoplasty in mildly obese patients (BMI 30-35 kg/m^2^): metabolic, biochemical and complication analysis at one year. In Vivo. (2015) 29:757–61. 26546533

[14] Rocic P. Comparison of cardiovascular benefits of bariatric surgery and abdominal lipectomy. Curr Hypertens Rep. (2019) 21:37. 10.1007/s11906-019-0945-8 30953254

[15] Sailon AM Wasserburg JR Kling RR Pasick CM Taub PJ. Influence of large-volume liposuction on metabolic and cardiovascular health: a systematic review. Ann Plast Surg. (2017) 79:623–30. 10.1097/SAP.0000000000001195 28737560

[16] Seretis K Goulis DG Koliakos G Demiri E. The effects of abdominal lipectomy in metabolic syndrome components and insulin sensitivity in females: a systematic review and meta-analysis. Metabolism. (2015) 64:1640–9. 10.1016/j.metabol.2015.09.015 26475176

[17] Seretis K Goulis DG Koliakos G Demiri E. Short- and long-term effects of abdominal lipectomy on weight and fat mass in females: a systematic review. Obes Surg. (2015) 25:1950–8. 10.1007/s11695-015-1797-1 26210190

[18] Ngaage LM Elegbede A Pace L Rosen C Tannouri S Rada EM. Review of insurance coverage for abdominal contouring procedures in the postbariatric population. Plast Reconstr Surg. (2020) 145:545–54. 10.1097/PRS.0000000000006513 31985657

[19] Altieri MS Yang J Park J Novikov D Kang L Spaniolas K. Utilization of body contouring procedures following weight loss surgery: a study of 37,806 patients. Obes Surg. (2017) 27:2981–7. 10.1007/s11695-017-2732-4 28600616

[20] Lotfi P Engdahl R. Concepts and techniques in postbariatric body contouring: a primer for the internist. Am J Med. (2019) 132:1017–26. 10.1016/j.amjmed.2019.02.048 30904509

[21] van der Beek ES van der Molen AM van Ramshorst B. Complications after body contouring surgery in post-bariatric patients: the importance of a stable weight close to normal. Obes Facts. (2011) 4:61–6. 10.1159/000324567 21372612 PMC6444757

[22] King WC Hinerman AS Belle SH Wahed AS Courcoulas AP. Comparison of the performance of common measures of weight regain after bariatric surgery for association with clinical outcomes. JAMA. (2018) 320:1560–9. 10.1001/jama.2018.14433 30326125 PMC6233795

[23] Sandvik J Hole T Klockner CA Kulseng BE Wibe A. High-frequency of computer tomography and surgery for abdominal pain after Roux-en-Y gastric bypass. Obes Surg. (2018) 28:2609–16. 10.1007/s11695-018-3223-y 29619755

[24] Gusenoff JA Messing S O'Malley W Langstein HN. Temporal and demographic factors influencing the desire for plastic surgery after gastric bypass surgery. Plast Reconstr Surg. (2008) 121:2120–6. 10.1097/PRS.0b013e31817081a3 18520904

[25] Cai A Maringa L Hauck T Boos AM Schmitz M Arkudas A. Body contouring surgery improves physical activity in patients after massive weight loss-a retrospective study. Obes Surg. (2020) 30:146–53. 10.1007/s11695-019-04145-3 31444775

[26] Monpellier VM Antoniou EE Mulkens S Janssen IMC van der Molen ABM Jansen ATM. Body image dissatisfaction and depression in postbariatric patients is associated with less weight loss and a desire for body contouring surgery. Surg Obes Relat Dis. (2018) 14:1507–15. 10.1016/j.soard.2018.04.016 30131312

[27] Soldin M Mughal M Al-Hadithy N. National commissioning guidelines: body contouring surgery after massive weight loss. J Plast Reconstr Aesthet Surg. (2014) 67:1076–81. 10.1016/j.bjps.2014.04.031 24909630

[28] Hunecke P Toll M Mann O Izbicki JR Blessmann M Grupp K. Clinical outcome of patients undergoing abdominoplasty after massive weight loss. Surg Obes Relat Dis. (2019) 15:1362–6. 10.1016/j.soard.2019.06.001 31296446

[29] de Vries CEE Kalff MC van Praag EM Florisson JMG Ritt M van Veen RN. The influence of body contouring surgery on weight control and comorbidities in patients after bariatric surgery. Obes Surg. (2020) 30:924–30. 10.1007/s11695-019-04298-1 31792701 PMC7347702

